# The Effect of Leucine Supplementation on Sarcopenia-Related Measures in Older Adults: A Systematic Review and Meta-Analysis of 17 Randomized Controlled Trials

**DOI:** 10.3389/fnut.2022.929891

**Published:** 2022-07-01

**Authors:** Yufei Guo, Xiaoya Fu, Qingjing Hu, Lihua Chen, Hui Zuo

**Affiliations:** ^1^School of Public Health, Suzhou Medical College of Soochow University, Suzhou, China; ^2^Department of Nutrition and Food Hygiene, School of Public Health, Nantong University, Nantong, China; ^3^Jiangsu Key Laboratory of Preventive and Translational Medicine for Geriatric Diseases, Suzhou Medical College of Soochow University, Suzhou, China

**Keywords:** leucine, sarcopenia, systematic review, meta-analysis, randomized controlled trials (RCTs), the elderly

## Abstract

**Background:**

The role of leucine in sarcopenia prevention remains unclear. We aimed to summarize the published data from randomized controlled trials (RCTs) to estimate the effect of leucine supplementation on sarcopenia-related measures in older adults.

**Methods:**

A systematic literature search was performed using the electronic databases PubMed, Embase, and Web of Science with restriction to randomized controlled trials design from January 1, 2009 to March 19, 2022. Sarcopenia-related measures included handgrip strength, total lean mass, gait speed, leg press, 6-min walk test, short-physical performance battery, timed up-and-go test and 30-s chair-stand test. Fixed- and random-effects meta-analysis models were used to generate pooled weighted mean differences (WMDs) and 95% CIs. Heterogeneity was examined in subgroup and sensitivity analyses. Publication bias assessments were performed.

**Results:**

A total of 17 RCTs enrolling 1418 subjects were identified. Leucine-isolated supplementation showed no effect on total lean mass (WMD = 0.03 kg, 95% CI: –0.51, 0.57, *P* = 0.917), handgrip strength (WMD = 1.23 kg, 95% CI: –0.58, 3.03, *P* = 0.183) and leg press (WMD = –1.35 kg, 95% CI: –7.46, 4.77, *P* = 0.666). However, leucine-combined supplementation including vitamin D showed a significant improvement in handgrip strength (WMD = 2.17 kg, 95% CI: 0.24, 4.10, *P* = 0.027) and gait speed (WMD = 0.03 m/s, 95% CI: 0.01, 0.05, *P* = 0.008).

**Conclusion:**

Leucine-isolated supplementation did not improve muscle mass and strength in elderly. However, leucine-combined supplementation including vitamin D exhibited a significant benefit for muscle strength and performance including handgrip strength and gait speed in older adults. A combination of nutritional supplements would be a viable option for improving sarcopenia.

## Introduction

Sarcopenia is a progressive loss of muscle mass, strength, and function (1), which usually develops with advanced age (2). The estimated prevalence of sarcopenia in people aged over 60 years was 10% according to a meta-analysis including 58404 individuals around the world (3). After the age of 60, the estimated muscle mass decreased at a rate of 3% per year, while the grip strength and gait speed decreased at a rate of 1.9–5.0% and 2.0–2.3% per year, respectively (4, 5). Sarcopenia was reported as one of the leading health issues in the older adults which could reduce the quality of life in the long term (6), and cause adverse health consequences including malnutrition (7), falls (8), disability, and even death (9). Although accumulating studies have focused on sarcopenia (10), there are still challenges in prevention and treatment of the disease. The homeostasis of amino acids has been increasingly suggested to be critical to maintaining muscle health (11).

L-leucine is an essential non-polar aliphatic, branched-chain amino acid (12), which activates the transducer of regulated cAMP response element-binding protein activity 1 (TORC1) in human skeletal muscle. The activation of TORC1 contributes to the initial stimulus of muscle protein synthesis, increasing the availability of amino acids through translation (13, 14). Leucine has a strong effect on energy and lipid metabolism (15). Increased energy expenditure and toxic lipids removal by increasing the prevalence and activity of leucine may be a promising therapeutic strategy to treat obesity and its consequent conditions (16, 17). Furthermore, some observational studies and randomized controlled trials (RCTs) have reported associations between leucine and muscle mass, muscle properties, and muscle functions (18–20).

Studies suggested that L-leucine supplementation was able to enhance muscle protein synthesis in the elderly (21–23). Some RCTs indicated that leucine could improve clinical indicators of sarcopenia in the elderly, including functional performance, and improve bone mineral-free lean tissue mass (12, 24). In contrast, several trials reported that prolonged leucine supplementation could not modulate body composition, muscle mass, and strength in elderly individuals (25–27). Inconsistent findings motivated a comprehensive systematic review and meta-analysis that evaluates the relationship between leucine and sarcopenia measures. Therefore, we summarized the latest evidence for the effect of leucine supplementation on sarcopenia measures in older adults based on published data from RCTs.

## Materials and Methods

### Literature Search

We searched the literature in the electronic bibliographic databases of PubMed, Embase, and Web of Science from January 1, 2009 to March 19, 2022 by following keywords or phrases: (“amino acid” OR “L-Leucine” OR “L-isomer Leucine” OR “leucine” OR “Leu”) AND (“RCT” OR “controlled trial” OR “randomized trial”) AND (“sarcopenia”). The detailed search strategies were listed in Supplementary Table 1. The search was restricted to human studies published in the English language with full text available.

### Selection of Articles

Screening of the literature and extraction of the data were done by XF and YG. First, we exported the literature from the database, and then we browsed every reference according to the title and abstract after removing the duplicate references. After deleting the irrelevant literature, we further browsed the full text of each article. Studies incorporated into the final analysis need to meet the following criteria: (1) were original investigations; (2) were randomized controlled trials; (3) the subjects were over 60 years old; (4) reported at least one diagnostic criterion for sarcopenia, including muscle mass (total lean mass), muscle strength (handgrip strength and leg press), physical performance (gait speed, short-physical performance battery, 6-min walk test, 30-s chair-stand test, and timed up-and-go test); (5) reported the doses of leucine.

### Data Extraction

The contents to be extracted included authors, year of publication, study design, sample size, mean age, gender, population, duration of follow-up, duration of intervention, leucine-isolated (yes/no), with/without vitamin D, physical exercise, type of leucine and dosage (g/day), muscle mass, muscle strength, and muscle performance outcomes. Study quality was assessed by the Modified Jadad Scale (scores ranged from 0 to 7).

### Statistical Analyses

Comparisons were made between the leucine-isolated/combined supplementation and control groups with reference to the difference in mean and standard deviation (SD) from baseline to final. We converted variances, standard errors, or confidence intervals to SD according to Cochrane Handbook when the data description form was not SD. Furthermore, we calculated the changes in mean and SD if only baseline and final data were available. Mean change values were calculated as the final mean minus the baseline mean. SD change values were estimated from the baseline and final SD using the following equation, derived from the Cochrane Handbook for Systematic Review of Interventions (28):


$$\begin{array}{c} SDchangescore=[{\left(SD_{\text{baseline}}\right)}^{2}+{\left(SD_{\text{final}}\right)}^{2}-2\\ \;\times correlation\times SD_{\text{baseline}}\times SD_{\text{final}}]{}^{1/2} \end{array}$$

In this equation, we used 0.8 as the assumed correlation (Use correlation coefficients obtained from studies according to the Cochrane Handbook).

The heterogeneity of the results was assessed using Cochran Q (Chi-square test) and *I*^2^ statistics. Statistical significance was set at *P* < 0.10 for Cochran Q test. When *I*^2^ > 50%, it was calculated by random effect. When *I*^2^ < 50%, the fixed effect was adopted. We performed subgroup analyses to explore the heterogeneity of the effect estimates based on modified Jadad score, vitamin D supplementation, physical activity, region of study and leucine-isolated/combined supplementation, dosage of leucine supplementation. All results were submitted for sensitivity analysis using the “remove-1” strategy. Publication bias was assessed by the Funnel plots and Egger’s regression model. Statistical significance was considered if the 95% CI did not contain 0. All the statistical analyses were performed using Stata version 15.1 (StataCorp, College Station, TX, United States).

## Results

### Study Selection

**FIGURE 1:**
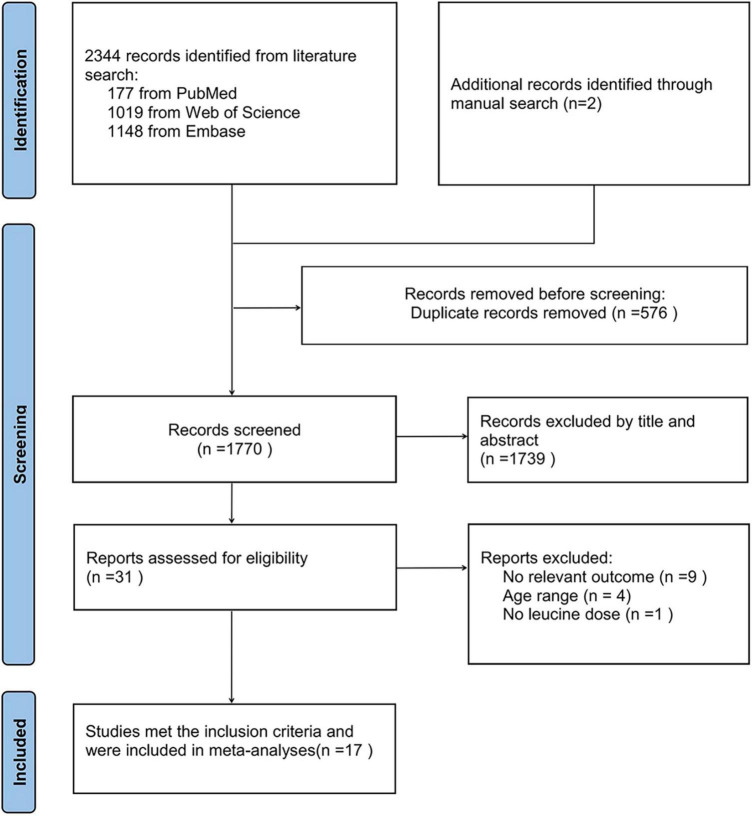
Flow chart of the study screening and selection process.

Figure 1 shows a flowchart of the study screening and selection process according to the Preferred Reporting Items for Systematic Reviews and Meta-Analyses (PRISMA) guidelines. We retrieved 2,346 records after searching the three databases, 177 from PubMed, 1,019 from Web of Science, 1,148 from Embase, and 2 through manual search. After removing duplicates (*n* = 576) and browsing the remaining articles (*n* = 1,770) by title and abstract, with three additional articles identified through manual searches, 31 records were screened out for further evaluation by carefully checking the full-length articles. Finally, we obtained 17 RCTs meeting the inclusion criteria. The quality of these studies is shown in Supplementary Table 2.

### Descriptive Characteristics of the Studies

Demographic data of the subjects and study characteristics of the included RCTs are summarized in Table 1. The total number of subjects from all the included studies was 1,418 (range of study sample size: 19,380). The mean age of the subjects ranged from 67.7 to 82.3. There were 11 studies that included men and women (12, 24, 25, 29–36), and six studies (26, 27, 37–40) included only male or female subjects. The duration of the intervention varied from 4 to 48 weeks. Subjects of nine studies (25, 26, 30, 33–36, 38, 39) took part in a controlled physical activity program. Total lean mass (TLM) was measured in nine studies (24, 26, 27, 30, 32, 34, 35, 37, 40). Handgrip strength was assessed in 11 studies (12, 26, 29–36, 40). Leg press was assessed in three studies (26, 27, 39). Gait speed was assessed in seven studies with eight treatment arms (29–33, 38, 40). Short-physical performance battery (SPPB) was assessed in six studies (25, 29, 31, 35, 36, 40). 6-min walk test (6-WT) was assessed in five studies with six treatment arms (24, 25, 33, 35, 39). 30-s chair-stand test (30-CST) was assessed in five studies with six treatment arms (24, 26, 29, 36, 40). Timed up-and-go test (TUG) was assessed in three studies (26, 29, 33).

**TABLE 1:** Study characteristics of the 17 included trials.

Author (year)	Country/Region	Mean age (Years)	Subjects (n)	Gender (% Female)	Duration of intervention (Weeks)	Leucine: g/day, Source	Leucine-isolated (Yes/No)	Vitamin D combined (Yes/No)	Outcome measures
**Group 1: all participants not physically exercised**									
Verhoeven et al. (37)	Netherlands	71	Healthy (29)	0	12	7.5, capsules	Yes	No	MM: total lean mass (TLM)
Leenders et al. (27)	Netherlands	71	Type 2 diabetes patients (57)	0	24	7.5, capsules	Yes	No	MM: total lean mass (TLM); MS: leg press
Kim et al. (38)	Japan	79.1	Elderly sarcopenic women (78) [Amino acid (n = 39), health education (n = 39)]	100	12	2.52, packets of powdered amino acid supplements	No	No	MP; gait speed
Ispoglou et al. (24)	England	71.6	Healthy (25)	56	12	3.175, 20% leucine capsules; 6.250, 40% leucine capsules	No	No	MM: total lean mass (TLM); MP: 30-s chair-stand test (30sec-CST), 6-min walk test (6-WT)
Martínez-Arnau et al. (12)	Spain	78.9	Men and women living in nursing homes (42)	69	13	6, powder	Yes	No	MS: handgrip strength
Murphy et al. (29)	Ireland	71.3	Men and women with low muscle mass and/or strength (69)	49.3	24	6.2, active supplements	No	No	MS: handgrip strength; MP: short-physical performance battery (SPPB), timed up-and-go test (TUG), gait speed, 30-s chair-stand test (30sec-CST)
Chanet et al. (40)	France	71	Healthy older men (24)	0	6	3, medical nutrition drink	No	Yes	MM: total lean mass (TLM); MS: handgrip strength; MP: short-physical performance battery (SPPB), 30-s chair stand test (30sec-CST), gait speed
Bauer et al. (31)	Germany	77.7	Sarcopenic older adults (380)	65.5	13	6, powder	No	Yes	MS: handgrip strength; MP: short-physical performance battery (SPPB), gait speed
Lin et al. (32)	Taiwan	73.1	Sarcopenic older adults (56)	28.6	12	1.2, a vitamin D- and leucine-combined whey protein supplement	No	Yes	MM: total lean mass (TLM); MS: handgrip strength; MP: gait speed
**Group 2: all participants physically exercised**									
Kim et al. (38)	Japan	79.1	Elderly sarcopenic women (77) [Exercise + amino acid (n = 38), exercise (n = 39)]	100	1	2.52, packets of powdered amino acid supplements	No	No	MP; gait speed
Amasene et al. (35)	Spain	82.3	Sarcopenic older adults (28)	50	12	0.857, protein supplement	No	No	MM: total lean mass (TLM); MS: handgrip strength; MP: 30-s chair-stand test (30sec-CST), 6-min walk test (6-WT), short-physical performance battery (SPPB)
Kirk et al. (25)	England	68	Previously untrained males, and females (46)	54.3	16	6.927, Vanilla flavored Whey Isolate Protein supplement	No	No	MP: short-physical performance battery (SPPB), 6-min walk test (6-WT)
Jacob et al. (39)	Canada	77.5	Pre/frail elderly women (19)	100	12	7.5, Powdered supplement	Yes	No	MS: leg press; MP: 6-min walk test (6-WT)
Roschel et al. (26)	Brazil	72	Pre/frail elderly women (44)	100	16	7.5, leucine	Yes	No	MM: total lean mass (TLM); MS: leg press, handgrip strength; MP: timed-up-and-go (TUG), 30-s chair-stand test (30sec-CST)
Yamamoto et al. (30)	Japan	72.7	Elderly with type 2 diabetes (36)	50	48	2.4, amino acid supplement	No	No	MM: total lean mass (TLM); MS: handgrip strength; MP: gait speed
Barichella et al. (33)	Italy	67.7	Patients with Parkinson’s disease or parkinsonism (150)	64	4	5.6, whey protein-based nutritional supplement	No	Yes	MS: handgrip strength; MP: 6-min walk test (6-WT), gait speed, timed up and go test (TUG)
Rondanelli et al. (34)	Italy	80.3	Sarcopenic older adults (130)	59.2	12	4, Dietary supplement	No	Yes	MM: total lean mass (TLM); MS: handgrip strength
Rondanelli et al. (36)	Italy	81	Sarcopenic older adults (140)	66	8	5.6, powder	No	Yes	MS: handgrip strength; MP: timed up and go test (TUG), 30-s chair stand test (30sec-CST), short-physical performance battery (SPPB)

*MM, muscle mass; MP, muscle performance; MS, muscle strength; SPPB, short-physical performance battery; TLM, total lean mass; TUG, timed up and go test; 30sec-CST: 30-s chair-stand test; 6-WT: 6-min walk test.*

### Muscle Mass

The effects of leucine-isolated/combined supplements on muscle mass were assessed by TLM. A total of nine RCTs (*n* = 438) reported TLM as an outcome indicator. No significant difference in TLM was observed in leucine-isolated/combined supplementation group compared with the placebo group (WMD = 0.29 kg, 95% CI: –0.06, 0.63, *P* = 0.102; Supplementary Figure 1).

### Muscle Strength

**FIGURE 2:**
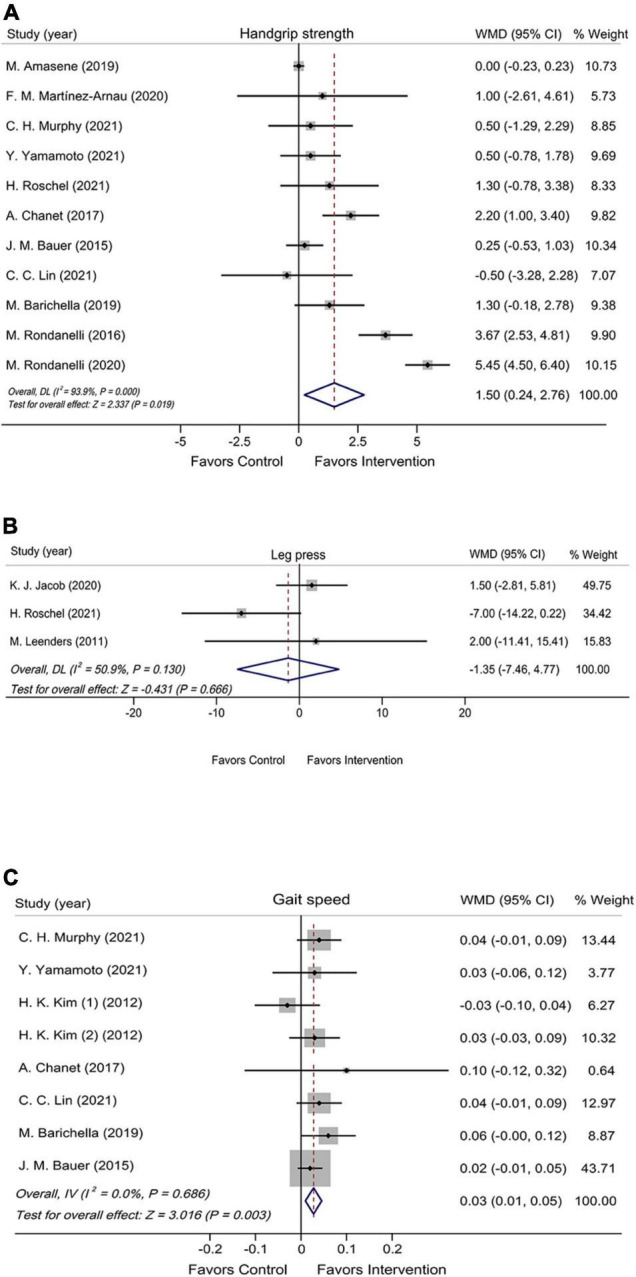
Forest plots assessing the effect of leucine-isolated/combined supplementation on handgrip strength **(A)**, leg press **(B)**, and gait speed **(C)**.

The effect of leucine-isolated/combined supplement on muscle strength was assessed by handgrip strength and leg press. Handgrip strength was assessed in 11 RCTs (n = 983), and it was significantly improved in the leucine-isolated/combined supplementation group compared with the control group (WMD = 1.50 kg, 95% CI: 0.24, 2.76, *P* = 0.019; Figure 2A). Leg press was assessed in three RCTs (n = 120), and no significant effect was observed for leucine-isolated supplement (WMD = –1.35 kg, 95% CI: –7.46, 4.77, *P* = 0.666; Figure 2B).

### Muscle Performance

Muscle performance was assessed by gait speed, SPPB, 6-WT, 30sec-CST, and TUG. The pooled results of seven RCTs (n = 772) showed that leucine-isolated/combined supplements significantly accelerated gait speed (WMD = 0.03 m/s, 95% CI: 0.01, 0.05, *P* = 0.003; Figure 2C), whereas the intervention had no effect on SPPB (WMD = 0.34 scores, 95% CI: –0.55, 1.24, *P* = 0.453; Supplementary Figure 2A) and 6-WT (WMD = –3.58 m, 95% CI: –13.31, 6.15, *P* = 0.470; Supplementary Figure 2B). Among the five RCTs (*n* = 280) and three RCTs (*n* = 246) assessed 30sec-CST and TUG, neither of which showed significant improvement after supplementation (30sec-CST: WMD = 2.50 times, 95% CI: –0.26, 5.25, *P* = 0.076; Supplementary Figure 3A); (TUG: WMD = –0.05 s, 95% CI: –0.56, 0.46, *P* = 0.847; Supplementary Figure 3B).

### Subgroup Analyses

**FIGURE 3:**
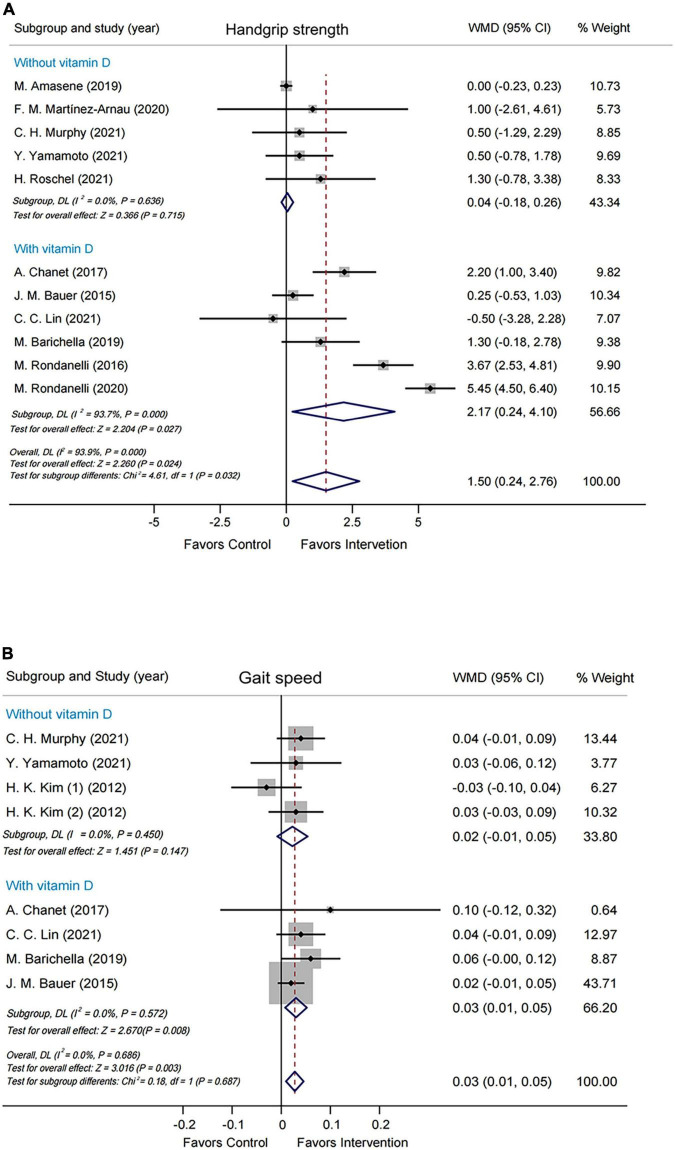
Forest plots assessing the effect of leucine supplementation on handgrip strength **(A)** and gait speed **(B)** by groups with or without vitamin D.

**FIGURE 4:**
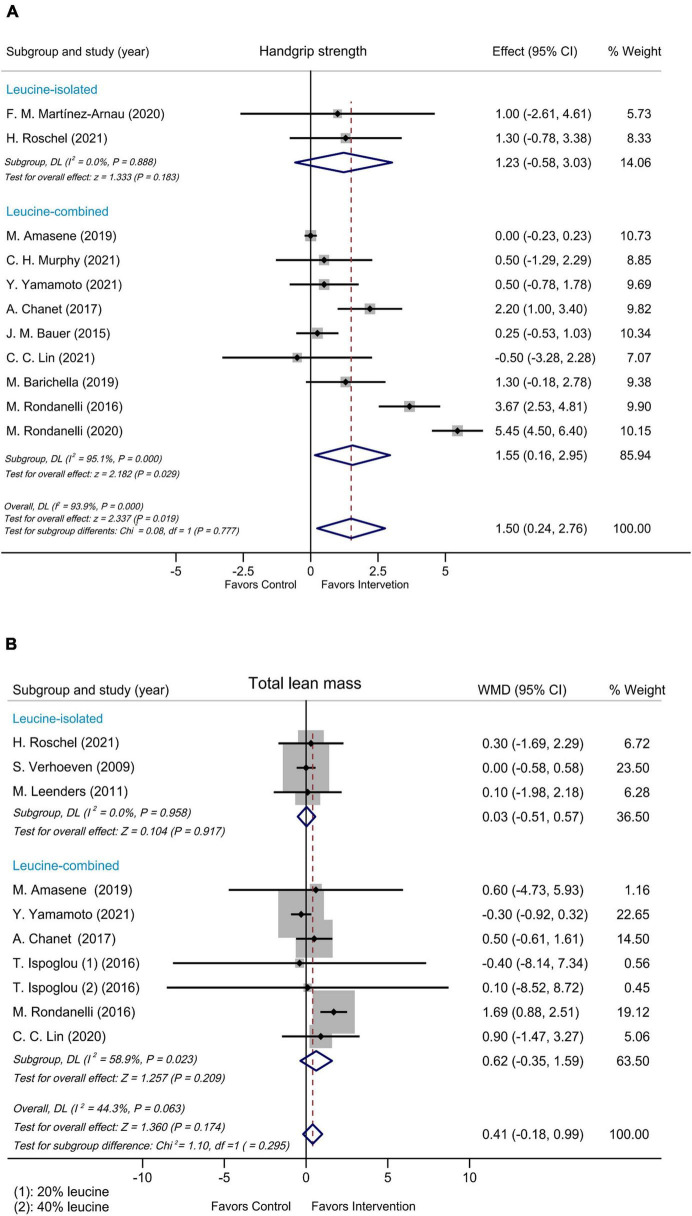
Forest plots assessing the effect on handgrip strength **(A)** and TLM **(B)** by leucine-isolated/combined supplementation.

Subgroup analyses were performed by modified Jadad score, presence/absence of vitamin D supplementation, physical activity, country/region of study, leucine-isolated/combined supplementation and dosage of leucine supplementation. Improvement in handgrip strength was observed in the studies where modified Jadad scores >3 (WMD = 1.79 kg, 95% CI: 0.32, 3.25, *P* = 0.017), but not in those studies with modified Jadad scores ≤ 3 (WMD = 0.32 kg, 95% CI: –0.84, 1.49, *P* = 0.584; Supplementary Figure 4). A significantly beneficial effect on handgrip strength was found in the group supplemented with vitamin D (WMD = 2.17 kg, 95% CI: 0.24, 4.10, *P* = 0.027) compared with the group without vitamin D (WMD = 0.04 kg, 95% CI: –0.18, 0.26, *P* = 0.715; Figure 3A). Besides, there was an improvement in handgrip strength in the leucine-combined group (WMD = 1.55 kg, 95% CI: 0.16, 2.95, *P* = 0.029), but not in the leucine-isolated group (WMD = 1.23 kg, 95% CI: –0.58, 3.03, *P* = 0.183; Figure 4A). In addition, gait speed was significantly improved among the studies from Europe and America (WMD = 0.03 m/s, 95% CI: 0.01, 0.05, *P* = 0.008; Supplementary Figure 5A), those with leucine-combined supplements containing vitamin D (WMD = 0.03 m/s, 95% CI: 0.01, 0.05, *P* = 0.008; Figure 3B) and those with supplementation doses ≥ 5 g/day (WMD = 0.03 m/s, 95% CI: 0.01, 0.05, *P* = 0.009; Supplementary Figure 5B). Additionally, there was no difference in TLM between the leucine-combined group (WMD = 0.62kg, 95% CI: –0.35, 1.59, *P* = 0.209) and the leucine-isolated group (WMD = 0.03 kg, 95% CI: –0.51, 0.57, *P* = 0.917; Figure 4B). Besides, no difference in handgrip strength and gait speed was observed when the subjects were stratified by physical activity. Also, when the subgroup analysis was based on the modified Jadad score, vitamin D supplementation, physical activity, country/region of study, and dosage of leucine supplementation, there was no difference in the other indicators of muscle mass, muscle strength and performance.

### Sensitivity Analysis and Publication Bias

Sensitivity analyses indicated that our findings were robust for TLM, handgrip strength, gait speed, 30sec-CST, TUG, SPPB, 6-WT, and leg press. Moreover, the symmetrical shape of the funnel plot indicated low publication bias (all p-values of Egger’s test > 0.1; data not shown).

## Discussion

Our findings based on 17 RCTs showed that leucine-isolated supplementation had no effect on total lean mass, handgrip strength and leg press, but leucine-combined supplementation including vitamin D could significantly improve handgrip strength and gait speed in older adults. In addition, we observed gait speed was improved for the intake of ≥5 g of leucine supplements and in those studies conducted among non-Asians.

After two previous meta-analyses in 2015 (41, 42), 14 newly published RCTs (12, 24–26, 29–36, 39, 40) were included in the present meta-analysis. Our updated findings indicated no significant difference in changes in lean body mass between the intervention and control group. The meta-analysis by B. Komar et al. included 16 studies testing leucine-combined supplements in a wider variety of participants, who were also frail, sarcopenic, and geriatric hospitalized. In that study, leucine-combined supplementation increased lean body mass (Mean difference = 0.99 kg, 95% CI: 0.43, 1.55), which was inconsistent with our findings. However, null effect of leucine on muscle strength was reported by B. Komar et al. (42), which was similar to our results. In addition, we found a significant improvement in handgrip strength after taking leucine-combined supplementation including vitamin D. Another one from Z. Xu et al included nine RCTs, including healthy participants and participants with cancer and type 2 diabetes, which showed no significant effect of leucine supplementation on lean body mass (41), which was consistent with our results. Studies by Z. Xu et al. have also shown that leucine supplementation increased the rate of muscle protein fraction synthesis (Standardized mean difference = 1.08, 95% CI: 0.5, 1.67), which was not analyzed in our meta-analysis due to the lack of references using the rate of muscle protein fraction synthesis as a primary or secondary measure end-point.

Leucine plays an important role in maintaining skeletal muscle metabolism. Muscle mass is regulated daily by muscle protein synthesis (MPS) and breakdown (MPB). However, aging disrupts the response of MPS to anabolic stimuli and corresponding protein balance. Leucine stimulates mTOR (a major regulator of protein synthesis) and MPS (43). Moreover, leucine can influence proteolysis by inhibiting associated catabolic transcription factors (e.g., FoxO3) (44). A previous study by Chae et al. has observed a positive correlation between daily leucine intake and skeletal muscle mass index in middle-aged individuals, skeletal muscle mass index increased by 0.29%, when each 1g/day increased in leucine (45). Similarly, the study by Lixandrão et al. showed there was a moderate and positive association between total daily leucine intake (g/day) and both quadriceps muscle cross-sectional area (β = 1.7) and maximum dynamic muscle strength (β = 2.4) (When the leucine dose changes by one unit, maximum dynamic muscle strength and muscle cross-sectional area change by β units) (46). However, in our meta-analysis, leucine-isolated supplementation showed no effect on total lean mass, handgrip strength and leg press. There are several possible explanations for this. First, it is possible that leucine-isolated supplements do have a relatively slight effect on muscle mass and muscle strength. Second, the effective dose of leucine may be largely reduced by *in vivo* metabolism. About 40% of leucine absorbed by muscle accumulates in intracellular free pools, 20% is incorporated into proteins, and 40% is oxidized (47). Third, potential confounding factors may bias the observed results. Moreover, the intervention doses in most of the included RCTs may not be at levels that produce significant effects. There was evidence that the daily protein intake of older adults was insufficient (48). In particular, the elderly tended to consume less animal protein (there was evidence that leucine was more abundant in animal protein than plant protein) (49, 50). Our subgroup analyses showed significant improvement in gait speed with leucine supplementation of 5 g or more. This may indicate a need for a higher dose of leucine intervention, as evidenced by Casperson et al.’s study that long-term leucine supplementation resulted in a higher MPS rate (22). In addition, Park et al. observed a positive correlation between leucine dose and grip strength. when each 1g/day increased in leucine, grip strength increased by 0.796 kg (quartiles 4) (51). This may mean that follow-up studies can set multiple leucine supplementation doses to explore the association between leucine supplementation doses and the risk of sarcopenia. In addition, if the population’s total protein intake is already enough, leucine supplementation may not provide additional benefits (52).

Our meta-analysis found that leucine-combined supplementation including vitamin D significantly improved handgrip strength and gait speed. Vitamin D plays an important role in maintaining the physiological function of skeletal muscle. Pfeifer et al. reported that vitamin D could improve muscle mass in the elderly (53), but some researchers reported no significant improvement in muscle mass or strength by vitamin D (54, 55). Moreover, a meta-analysis of the effect of vitamin D monotherapy on sarcopenia showed that vitamin D supplementation had no effect on muscle mass (appendicular lean mass) and muscle strength (handgrip strength) and muscle performance parameters except SPPB (56). The leucine-combined supplementation including vitamin D might be more effective than leucine- or vitamin D-isolated supplements. The reason remains unclear, but it was hypothesized that both vitamin D and leucine inhibit atrophy-related transcription factors, and stimulate mTOR to promote protein synthesis (13, 14, 57, 58).

The present study has several limitations. First, heterogeneity existed among the included RCTs, which might be due to different study populations, leucine dosing, and regimens, interventions, variability in study design, residual bias, etc. Therefore, we used a random-effect model for subgroup analyses and meta-regression analysis for items with heterogeneity > 50%. Heterogeneity was not addressed in this section that might arise from differences in inclusion and exclusion criteria, patients’ baseline risk profiles, different brands of leucine, or differences in methodological quality. Second, few studies used leucine-isolated interventions, and heterogeneity in trial design and subjects might have influenced the results. Also, it might lead to poor representativeness of the samples and affect the extrapolation of results. Therefore, more RCTs are needed to address whether leucine supplements are effective to reduce sarcopenia in older adults. Third, no strictly control or measure of the leucine content in daily food intake of the subjects was taken in the RCTs that we included. Fourth, the cut-off values for the diagnosis of sarcopenia in men and women are different. The same effect size may represent different degrees of measures change in men and women. Our meta-analysis could not present results in men and women separately due to the lack of data. Fifth, we might not be able to eliminate the effect of physical exercise on sarcopenia-related measures. However, the subjects in the intervention and control groups in all the included RCTs either had no exercise or had exercise at the same frequency and intensity. Therefore, we assume that the change in outcome measures was not attributed to physical exercise.

## Conclusion

Leucine-isolated supplementation had no significant effect on total lean mass, handgrip strength and leg press in older adults. Instead, leucine-combined supplementation including vitamin D could significantly improve muscle strength and muscle performance. More experimental studies are needed to clarify and better understand the effect of leucine supplementation on sarcopenia.

## Data Availability Statement

The raw data supporting the conclusions of this article will be made available by the authors, without undue reservation.

## Author Contributions

YG and XF performed the literature search, extracted and analyzed the data, and drafted the manuscript. QH assisted the literature search and data analyses. HZ conceptualized and supervised the work, and had primary responsibility for the final content of the manuscript. QH, LC, and HZ critically revised the manuscript for important intellectual content. All authors reviewed and approved the final manuscript.

## Conflict of Interest

The authors declare that the research was conducted in the absence of any commercial or financial relationships that could be construed as a potential conflict of interest.

## Publisher’s Note

All claims expressed in this article are solely those of the authors and do not necessarily represent those of their affiliated organizations, or those of the publisher, the editors and the reviewers. Any product that may be evaluated in this article, or claim that may be made by its manufacturer, is not guaranteed or endorsed by the publisher.

## Supplementary Material

Supplementary Figure 1 Forest plots assessing the effect of leucine supplementation on total lean mass.

Supplementary Figure 2 Forest plots assessing the effect of leucine supplementation on SPPB **(A)** and 6-WT **(B)**.

Supplementary Figure 3 Forest plots assessing the effect of leucine supplementation on 30sec-CST **(A)**, TUG **(B)**.

Supplementary Figure 4 Forest plots assessing the effect of leucine supplementation on handgrip strength by modified Jadad score ≤3 and modified Jadad score >3.

Supplementary Figure 5 Forest plots assessing the effect of leucine supplementation on gait speed by Asian countries and Europe and America **(A)** and doses ≥5 g/day and <5 g/day **(B)**.

Supplementary Table 1 Literature search strategy for meta-analysis.

Supplementary Table 2 Quality assessment of the included studies according to modified Jadad score.
