# Supplementary figures and images for: The Effect of Leucine Supplementation on Sarcopenia-Related Measures in Older Adults: A Systematic Review and Meta-Analysis of 17 Randomized Controlled Trials
Source: Front Nutr. 2022 Jul 1;9:929891. doi: 10.3389/fnut.2022.929891 (PMC9284268; doi:10.3389/fnut.2022.929891)

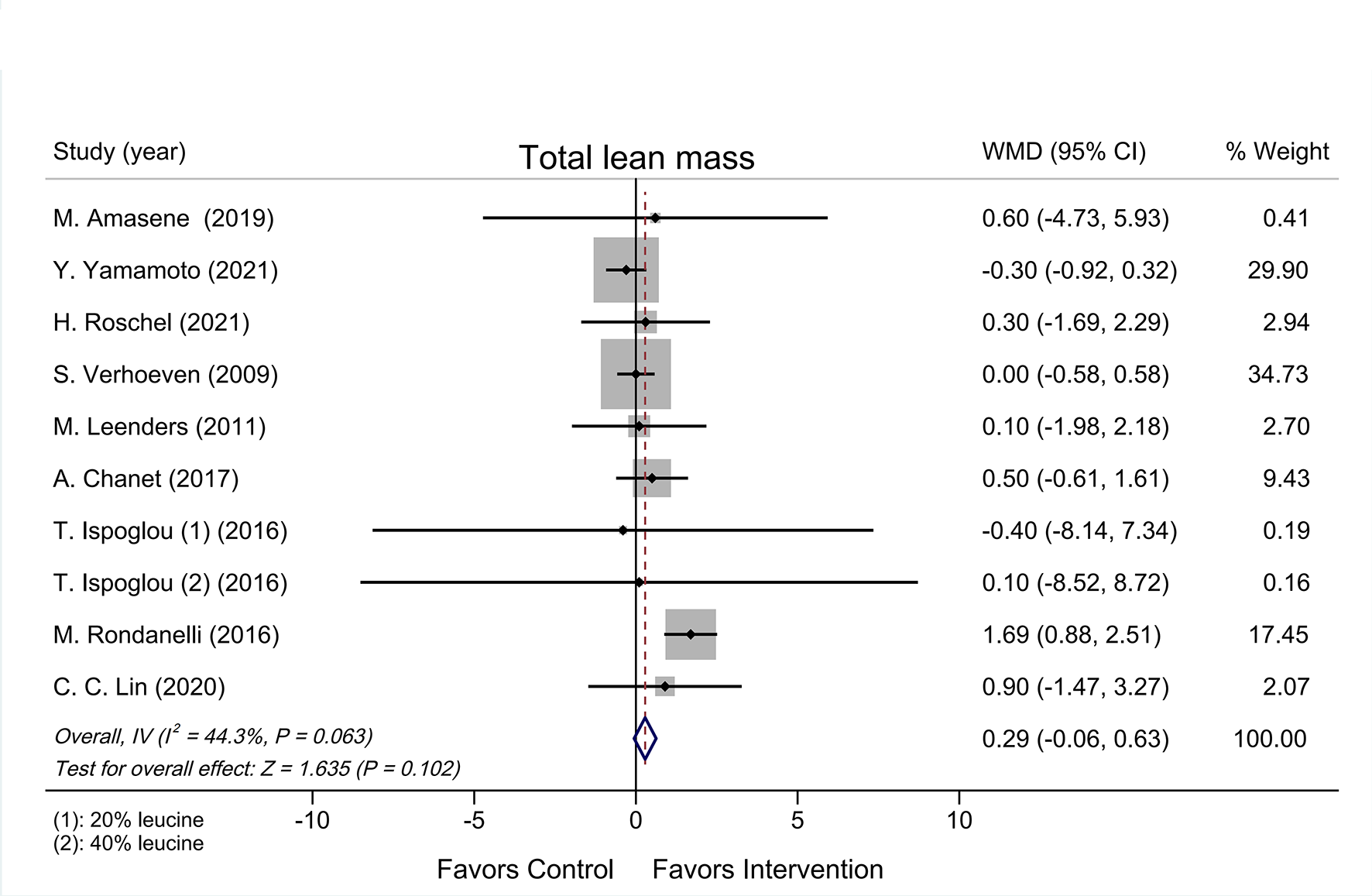

Supplement: Supplementary Figure 1 — Forest plots assessing the effect of leucine supplementation on total lean mass. [file Image_1.TIF]

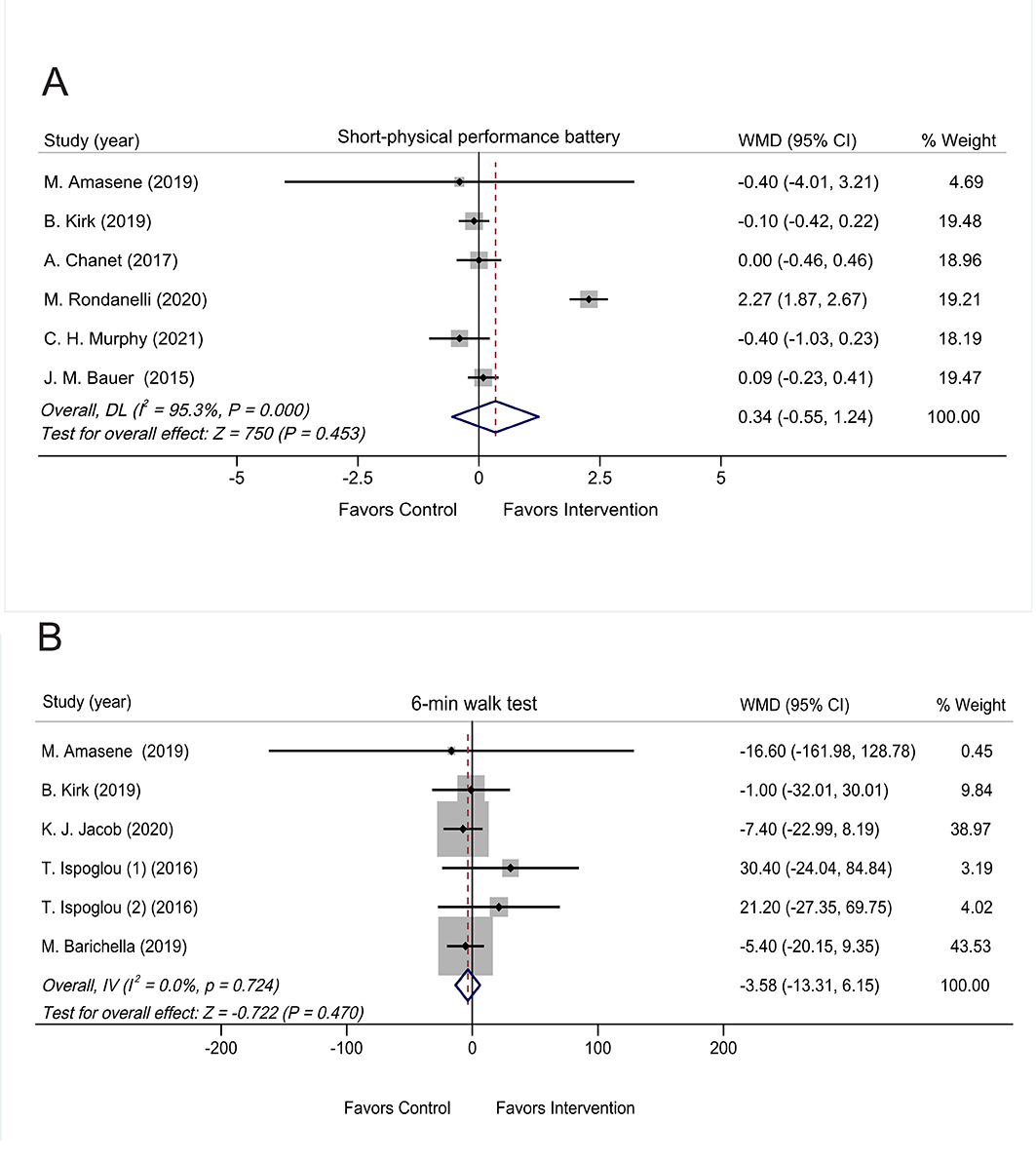

Supplement: Supplementary Figure 2 — Forest plots assessing the effect of leucine supplementation on SPPB (A) and 6-WT (B). [file Image_2.TIF]

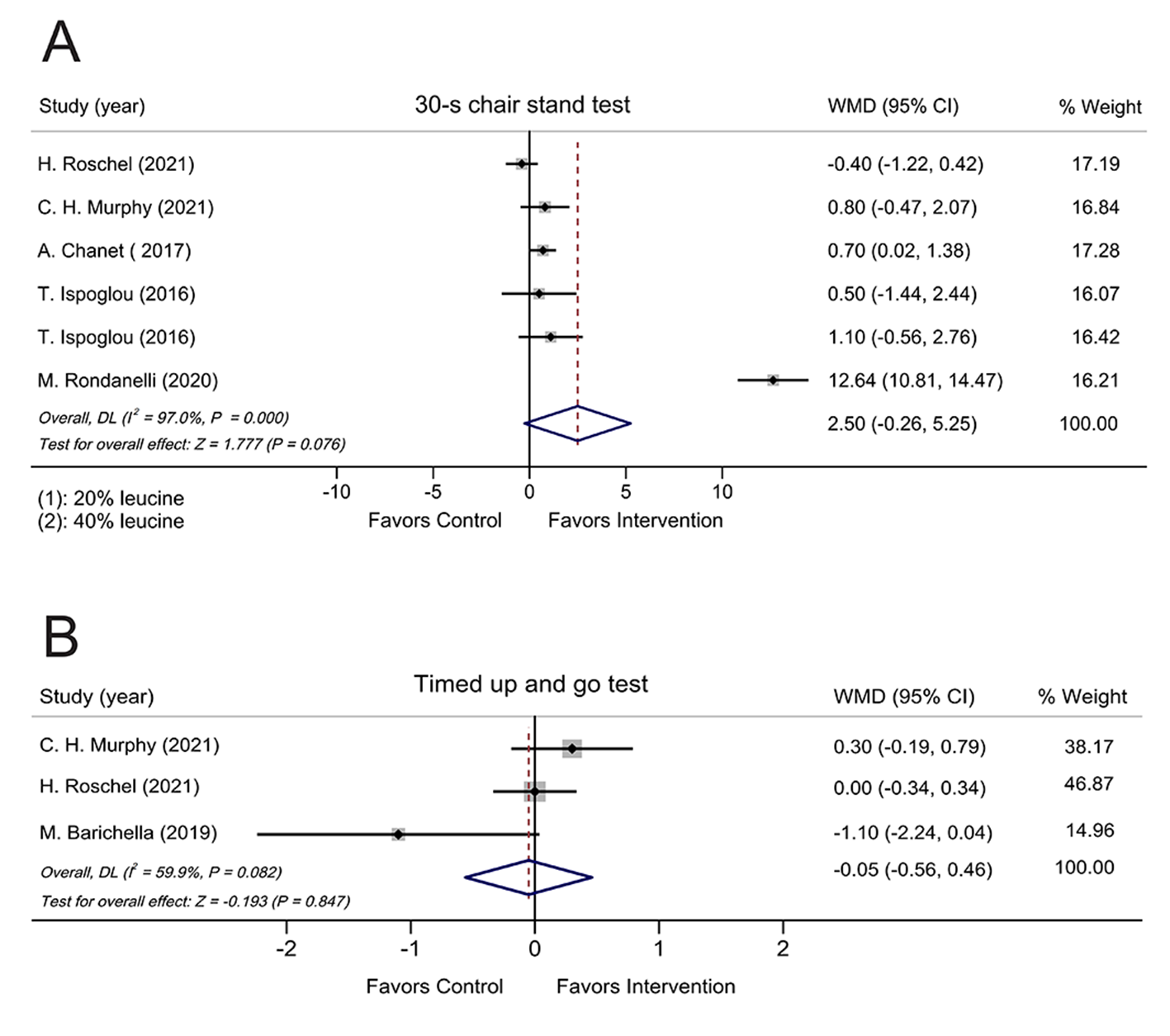

Supplement: Supplementary Figure 3 — Forest plots assessing the effect of leucine supplementation on 30sec-CST (A), TUG (B). [file Image_3.TIF]

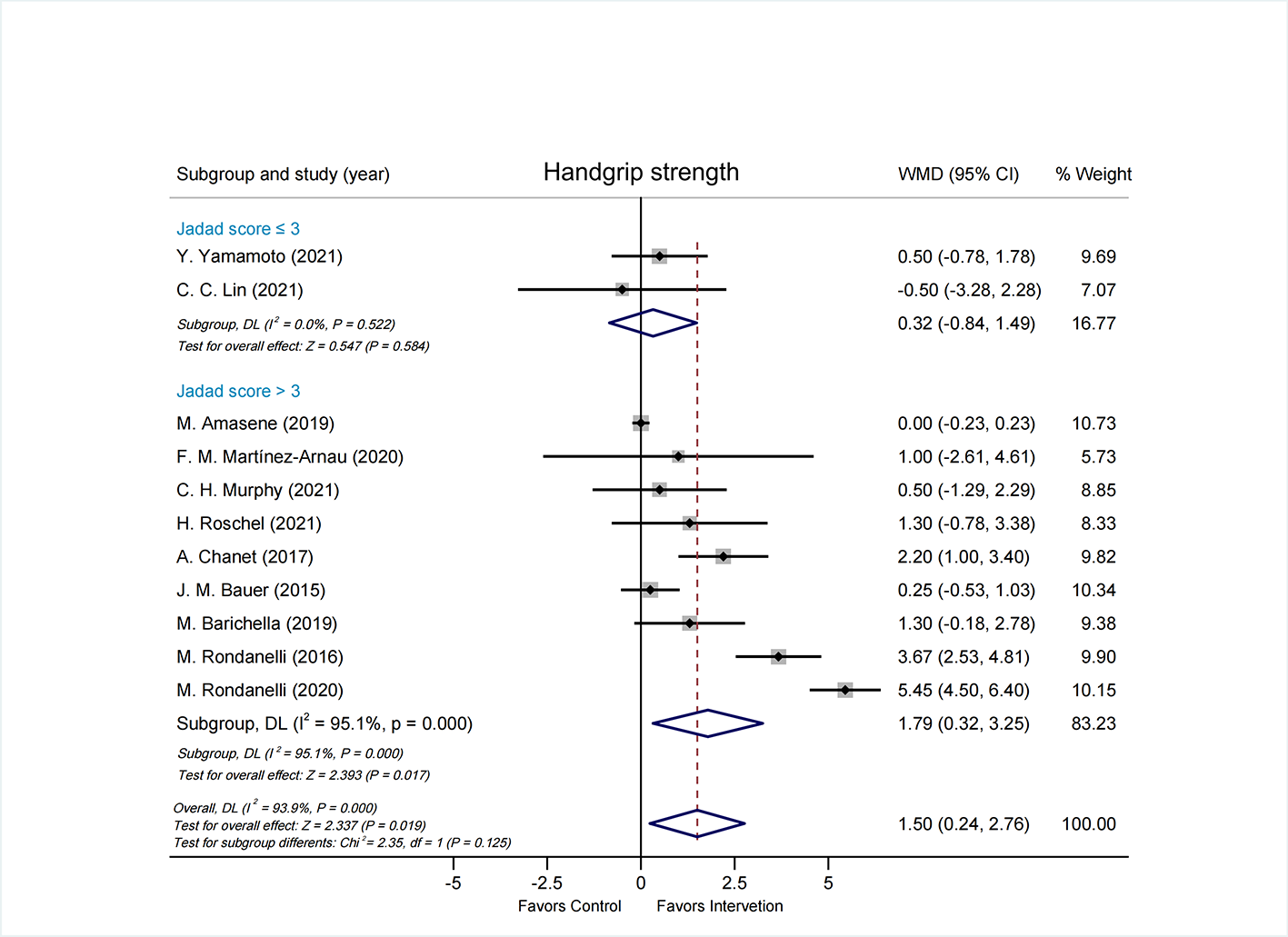

Supplement: Supplementary Figure 4 — Forest plots assessing the effect of leucine supplementation on handgrip strength by modified Jadad score ≤3 and modified Jadad score >3. [file Image_4.TIF]

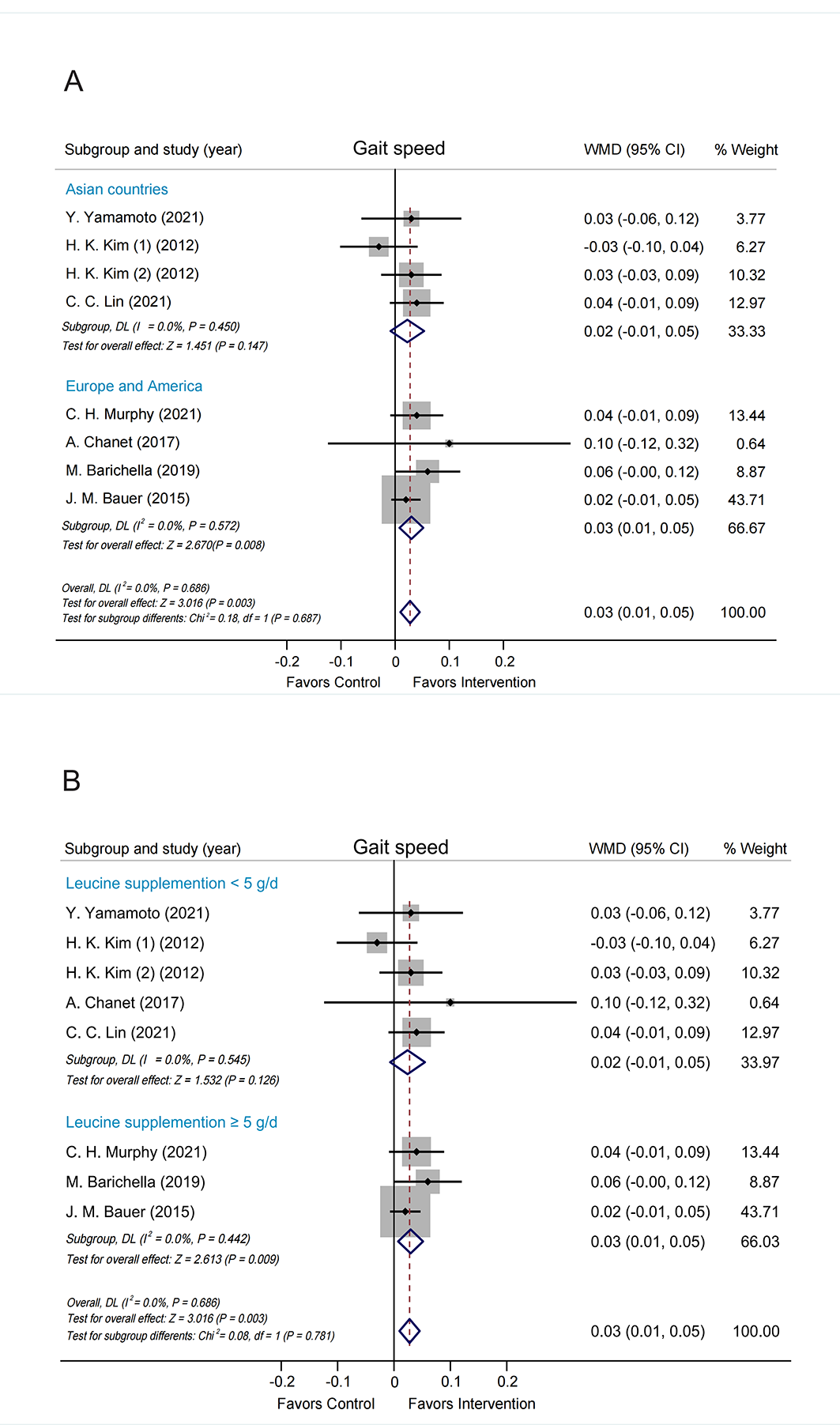

Supplement: Supplementary Figure 5 — Forest plots assessing the effect of leucine supplementation on gait speed by Asian countries and Europe and America (A) and doses ≥5 g/day and <5 g/day (B). [file Image_5.TIF]
