# Supplementary material for: The Effect of Leucine Supplementation on Sarcopenia-Related Measures in Older Adults: A Systematic Review and Meta-Analysis of 17 Randomized Controlled Trials
Source: Front Nutr. 2022 Jul 1;9:929891. doi: 10.3389/fnut.2022.929891 (PMC9284268; doi:10.3389/fnut.2022.929891)
Supplement: Supplementary Table 1 — Literature search strategy for meta-analysis. [file Table_1.DOCX]

**Supplementary table 1. Literature search strategy for meta-analysis.**

| **#1: Search “L-Leucine”[Mesh] OR “amino acid”[tiab] OR “L-isomer Leucine”[tiab] OR” leucine”[tiab] OR “Leu”[tiab];**  **#2: Search “Sarcopenia”;**  **#3: Search “RCT” OR “controlled trial” OR “randomized trial”;**  **#4: Search #1 AND #2 AND #3.** |
| --- |
