# Supplementary material for: The Effect of Leucine Supplementation on Sarcopenia-Related Measures in Older Adults: A Systematic Review and Meta-Analysis of 17 Randomized Controlled Trials
Source: Front Nutr. 2022 Jul 1;9:929891. doi: 10.3389/fnut.2022.929891 (PMC9284268; doi:10.3389/fnut.2022.929891)
Supplement: Supplementary Table 2 — Quality assessment of the included studies according to modified Jadad score. [file Table_2.DOCX]

**Supplementary Table 2.** Quality assessment of the included studies according to Modified Jadad Score

| Author (year) | Randomization | Allocation concealment | Blinding | Withdrawals and dropouts | Modified Jadad Score |
| --- | --- | --- | --- | --- | --- |
| S. Verhoeven (2009) (38) | 1 | 0 | 2 | 1 | 4 |
| M. Leenders (2011) (27) | 1 | 1 | 2 | 1 | 5 |
| H. K. Kim (2012) (39) | 2 | 2 | 0 | 1 | 5 |
| T. Ispoglou (2016) (24) | 1 | 1 | 2 | 0 | 4 |
| M. Amasene (2019) (36) | 1 | 1 | 2 | 1 | 5 |
| B. Kirk (2019) (25) | 1 | 1 | 1 | 1 | 4 |
| K. J. Jacob (2020) (40) | 2 | 1 | 1 | 1 | 5 |
| F. M. Martínez-Arnau (2020) (12) | 1 | 1 | 2 | 1 | 5 |
| C. H. Murphy (2021) (29) | 2 | 2 | 2 | 1 | 7 |
| H. Roschel (2021) (26) | 1 | 1 | 2 | 0 | 4 |
| Y. Yamamoto (2021) (30) | 1 | 1 | 0 | 1 | 3 |
| A. Chanet (2017) (41) | 1 | 1 | 2 | 1 | 5 |
| J. M. Bauer (2015) (32) | 2 | 2 | 2 | 1 | 7 |
| C. C. Lin (2021) (33) | 1 | 0 | 0 | 0 | 1 |
| M. Barichella (2019) (34) | 1 | 1 | 2 | 1 | 5 |
| M. Rondanelli (2016) (35) | 2 | 1 | 2 | 0 | 5 |
| M. Rondanelli (2020) (37) | 1 | 1 | 2 | 1 | 5 |
